# Resection of high-grade glioma involving language areas assisted by multimodal techniques under general anesthesia: a retrospective study

**DOI:** 10.1186/s41016-023-00340-5

**Published:** 2023-09-11

**Authors:** Meng Cui, Yukun Liu, Chunhui Zhou, Hewen Chen, Xin Gao, Jiayu Liu, Qingbao Guo, Bing Guan, Xiaodong Ma

**Affiliations:** 1 Department of Emergency, the Sixth Medical Center, Chinese People’s Liberation Army General Hospital, Beijing, China; 2 Department of Neurosurgery, the First Medical Center, Chinese People’s Liberation Army General Hospital, Beijing, China; 3 Department of Neurosurgery, Chinese Air Force Medical Center, Beijing, China; 4 Department of Neurosurgery, the Sixth Medical Center, Chinese People’s Liberation Army General Hospital, Beijing, China; 5 Department of Health Economics, the First Medical Center, Chinese People’s Liberation Army General Hospital, Beijing, China

**Keywords:** High-grade glioma, Language, Multimodal techniques, Intraoperative MRI, General anesthesia

## Abstract

**Background:**

Multimodal techniques-assisted resection of glioma under general anesthesia (GA) has been shown to achieve similar clinical outcomes as awake craniotomy (AC) in some studies. In this study, we aim to validate the use of multimodal techniques can achieve the maximal safe resection of high-grade glioma involving language areas (HGILAs) under GA.

**Methods:**

HGILAs cases were reviewed and collected between January 2009 and December 2020 in our center. Patients were separated into multimodal group (using neuronavigation, intraoperative MRI combined with direct electrical stimulation [DES] and neuromonitoring [IONM]) and conventional group (neuronavigation alone) and clinical outcomes were compared between groups. Studies of HGILAs were reviewed systematically and the meta-analysis results of previous (GA or AC) studies were compared with our results.

**Results:**

Finally, there were 263 patients in multimodal group and 137 patients in conventional group. Compared to the conventional group, the multimodal group achieved the higher median EOR (100% versus 94.32%, *P* < 0.001) and rate of gross total resection (GTR) (73.8% versus 36.5%, *P* < 0.001) and the lower incidence of permanent language deficit (PLD) (9.5% versus 19.7%, *P* = 0.004). The multimodal group achieved the longer median PFS (16.8 versus 10.3 months, *P* < 0.001) and OS (23.7 versus 15.7 months, *P* < 0.001) than the conventional group. The multimodal group achieved a higher rate of GTR than the cohorts in previous multimodal studies under GA and AC (73.8% versus 55.7% [95%CI 32.0–79.3%] versus 53.4% [35.5–71.2%]). The multimodal group had a lower incidence of PLD than the cohorts in previous multimodal studies under GA (9.5% versus 14.0% [5.8–22.1%]) and our incidence of PLD was a little higher than that of previous multimodal studies under AC (9.5% versus 7.5% [3.7–11.2%]). Our multimodal group also achieved a relative longer survival than previous studies.

**Conclusions:**

Surgery assisted by multimodal techniques can achieve maximal safe resection for HGILAs under GA. Further prospective studies are needed to compare GA with AC for HGILAs.

**Supplementary Information:**

The online version contains supplementary material available at 10.1186/s41016-023-00340-5.

## Background

High-grade glioma (HGG) is a high invasive type of gliomas, with an annual incidence of 0.78–4.24 per 100,000 [1]. Surgical resection is still the main method of HGG treatment, and the increment of extent of resection (EOR) has been confirmed to prolong survival of patients with HGG in many studies [2, 3]. However, more postoperative neurological deficits may be caused by more aggressive removal of tumor. Therefore, the surgical goal of HGG involving eloquent areas is maximal safe resection [4].

To achieve this goal, the application of direct electrical stimulation (DES) in neurosurgery was first proposed by Förster in 1929, and Ojemann and Berger established brain mapping techniques in glioma surgery using DES [5, 6]. At present, mapping by DES combined with intraoperative neuromonitoring (IONM) has been masterly applied in the resection of glioma. Because language and other advanced brain functions cannot be mapped and monitored according to the changes in evoked potentials directly, so awake craniotomy (AC) was developed and applied for the resection of glioma [7, 8]. However, this method has inherent shortcomings; for example, invasive stimulation, prolonged operation duration, increased risk of intraoperative seizures, bleeding, edema and anesthesia, and intraoperative tasks under AC cannot be completed in children or patients with preoperative severe neurological deficits [4, 9].

With the development of neuroimaging, eloquent area localization has become possible under GA. Task-based (tb-fMRI) or resting-state functional MRI (rs-fMRI), diffusion tensor imaging (DTI), and other imaging modes can be combined with neuronavigation, which can map the language areas (Broca area, Wernicke area, arcuate tract [AT], inferior occipito-frontal tract, etc.) in a noninvasive way [10]. These technologies also have some inherent shortcomings. Because they are based on preoperative imaging, intraoperative brain drift will lead to inaccurate localization [11]. MRI is also affected by many factors, such as the choice of the region of interest (ROI), signal-to-noise ratio, fractional anisotropy [FA]), and artifacts, etc. [12]. In order to increase the accuracy of neuronavigation based on preoperative multimodal imaging in localizing language areas, many techniques was developed, such as optimization of reconstruction algorithm and combination of tb-fMRI and rs-fMRI. In addition, we can overcome brain drift defects and increase EOR by using intraoperative MRI (iMRI) and other intraoperative imaging techniques [13]. Therefore, these multimodal techniques can help surgeon achieve maximal safe resection of HGG involving language areas (HGILAs) under GA [14].

The choice of GA or AC for the resection of HGILAs is still controversial among studies. Many studies advocated AC because of its superior EOR, language protection and survival compared with GA [15, 16]. However, more and more studies proved that multimodal techniques-assisted surgery under GA can achieve similar clinical outcomes as under AC for HGILAs [17, 18]. We had used iMRI to remove HGILAs since 2009 and developed our experience of surgery assisted by multimodal techniques under GA. In this study, by analyzing the outcomes of patients with HGILAs, we aimed to validate the use of multimodal techniques in surgery can achieve the goal of maximal safe resection under GA without causing more language deficit and prolong the survival of HGILAs.

## Methods

### Patient selection

Data of patients with HGILAs were retrospectively collected from electronic medical records in our center from January 2009 to December 2020. This study was approved by the ethics committee of Chinese People’s Liberation Army General Hospital. Anonymous data of patients were included according to the following inclusion criteria: (1) supratentorial HGG confirmed by pathology [19, 20]; (2) patients > 6 years old; (3) the distance ≤ 2 cm between the tumor and traditional language regions (Broca area/Wernicke area/dorsal premotor cortex and/or arcuate fasciculus) on preoperative MRI [21–24]; (4) resection assisted by neruonavigation alone or by multimodal techniques under GA; (5) pre/postoperative language function were assessed completely. The patients were excluded according to the criteria: (1) infratentorial HGG; (2) under 6 years old; (3) resection under AC; (4) biopsy alone (5) lost to follow-up.

### Patient grouping

The patients were divided into the conventional group (neuronavigation alone), and multimodal group (combined use of neuronavigation, iMRI, DES/IONM).

### Preoperative variables

Preoperative variables included age, sex, symptoms, aphasia quotient (AQ) by Western Aphasia Battery testing (AQ ≥ 93.8 and < 93.8 were defined as normal and aphasia, respectively) [25–27], occurrence of seizures and KPS to assess patients’ general functional status.

Tumor-related variables included location, recurrent tumor or not, volume (cm^3^), language cortices invaded or not, nearest distance to language areas (cortices or tracts) (mm), histopathology, molecular pathological findings. Since January 2016, patients with glioma have been commonly recommended for molecular testing. If the tumor was near language area but did not invaded it directly, the nearest distance was between the edge of tumor and language area. If the tumor invaded it directly, the nearest distance was 0 mm.

### Outcome variables

The outcome variables included EOR, postoperative 3-month/6-month AQ, and KPS, other surgery-related complications (hemorrhage, ischemia, intracranial infection, severe brain edema, etc.), postoperative seizures and their control, postoperative radiotherapy and cycles of TMZ chemotherapy, progression-free survival (PFS) and overall survival (OS). Language deficit was defined as a postoperative AQ less than the preoperative AQ at different time points.

### Preoperative MRI scanning and surgical plan

Preoperative MRI of patients were performed on a 1.5-T scanner (Siemens Espree, Erlangen, Germany). The imaging sequences and parameters were consistent with previous studies of our center [28]. During the BOLD-fMRI scanning, language tasks were performed by patients, including “picture naming”, “number counting”, and “word/sentence making”. The MRI data were imported into the Brainlab software, preoperative surgical plan was made by surgeon using iPlan 3.0. The iPlan of Brainlab (Feldkirchen, Germany) was used to measure tumor volume and distance. The iMRI or MRI within 48 h after surgery was performed to assess EOR. Gross total resection (GTR) was defined as EOR = 100% in this study.

### Surgical process assisted by multimodal techniques under GA

The surgeries with iMRI scanning were completed in a special operating room compatible with iMRI. GA used intravenous and volatile mixed anesthesia for all patients. The MRI data were imported into the Brainlab software. The Elements Image Fusion module of iPlan 3.0 was used to carry out all the imaging sequence fusions. The ROI was delineated by a board-certified neuroradiologist with 8 years of experience and a surgeon. The “SmartBrush” module of iPlan was used to delineate the tumor and reconstruct its 3D image. The delineation of HGG was performed on T1C images or T2/T2 FLAIR images. The language cortices was delineated according to brain functional anatomy and activated regions of BOLD-fMRI; then based on these delineated seed areas, the language tracts were reconstructed. FA was set as 0.12–0.16, length of pyramidal tract (PT) was set as 50–60 mm, arcuate tract was set as 30–40 mm and inferior occipito-frontal tract was set as 50–60 mm. MR angiography and venography were fused with other MR sequences to reconstruct vascular images when the tumor was close to important vessels. Finally, the surgical plan data were imported into neuronavigation.

After GA was completed, the head was fixed on the head holder. The navigation reference frame was fixed on one side of the head holder. The patient’s head and face were scanned with a laser indicator Z-touch® to achieve noncontact surface registration. After registration, we designed the surgical approach according to the location of the tumor and eloquent areas. Meanwhile, a neurophysiologist with 10 years of experience placed the needle electrodes into the patient’s scalp and limbs and connected them with an IONM system (Endeavor CR system, Nicolet®, USA). Then, the reference frame was removed, and a sterile frame was replaced after the surgical field was sterilized and covered by a sterile towel. After the bone flap was removed and the dura was opened, the cortices were exposed and the tumor was started to be removed.

**Fig. 1:**
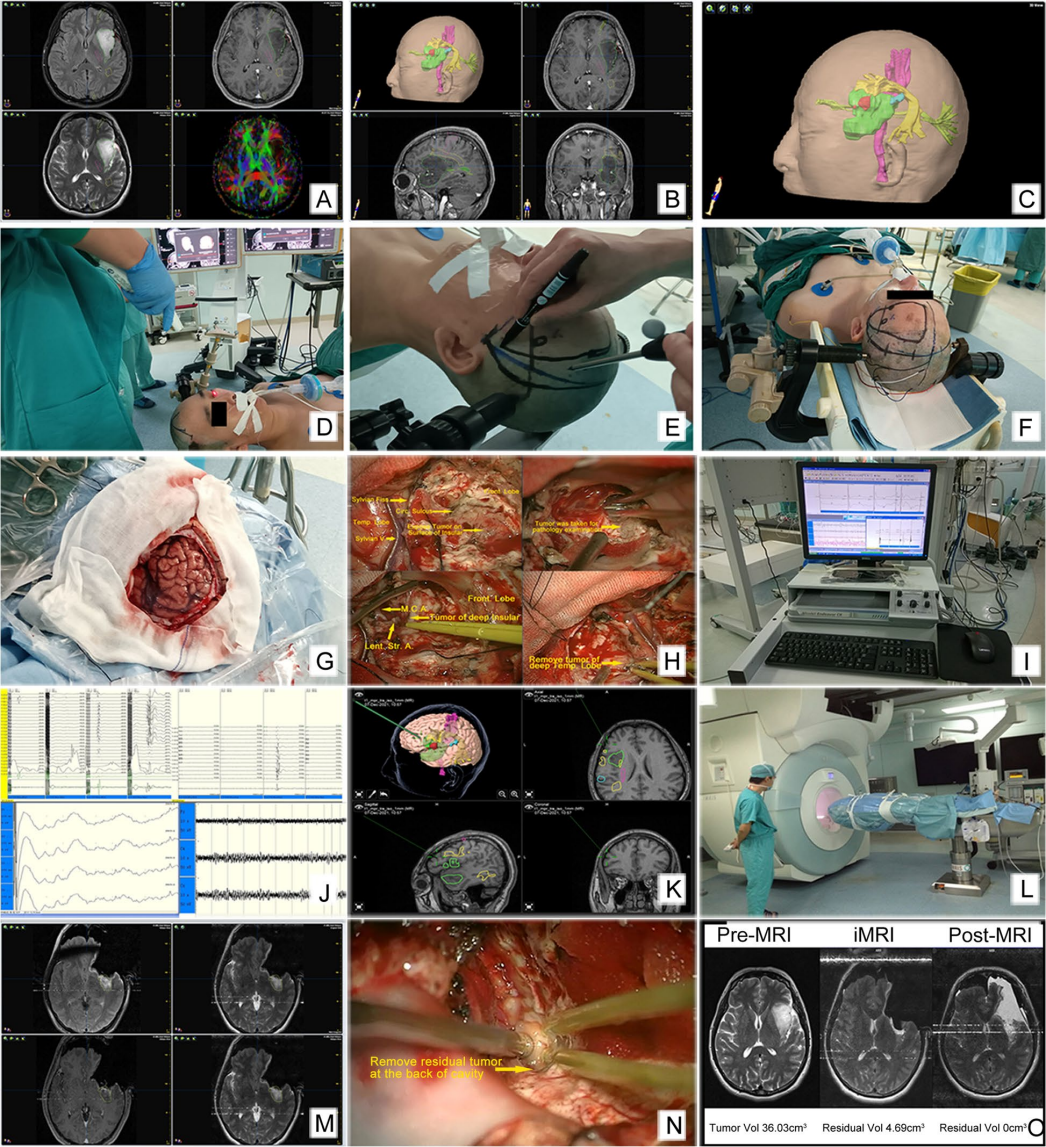
Surgical plan, approach design and process of GA. The patient was a 48-year-old woman with no preoperative symptoms and a KPS of 100. **A** Preoperative multimodal MRI showed a lesion in the left frontotemporal and insula lobes. Upper and lower left: Hyperintensity on T2 and FLAIR. Upper right: No obvious enhancement was found on T1C. Lower right: DTI showed that the lesion was close to the pyramidal tract, language cortices and tracts. **B**, **C** Surgical plan of neuronavigation showing the tumor (green), Broca area (red), Wernicke area (blue), PT (pink), AT (yellow) and inferior occipito-frontal tract (light green). Measurements: tumor volume, 36.03 cm^3^; shortest distance to PT, 3.8 mm; shortest distance to AT, 0 mm. **D** Registration by scanning the patient’s head and face with a laser indicator Z-touch®. **E** The surgeon designed the surgical approach and incision according to the guidance of the navigation probe and screen. **F** Needle electrodes were placed into the patient’s scalp and limbs for IONM. **G** Craniotomy through the pterion approach exposed the frontotemporal cortex and sylvian fissure. **H** Removed tumor under a microscope and guided by neuronavigation. Upper left: the avoided Broca area and part of the operculum were removed to expose the tumor of the insular lobe. Upper right: tumor sample was taken for pathology examination. Lower left: Removed tumor in the deep part insular to the surface of the putamen; the MCA and its branches were protected. Lower right: removed tumor of the deep part of the temporal lobe. **I** IONM system, MEP induced by transcranial stimulation compared with MEP at baseline. Changes in MEP, SEP, and current intensity by DES can locate the PT. **J** Intracranial MEP (upper left), MEP induced by DES (upper right), SEP (lower left), and EEG (lower right). IONM showed that the amplitude of the terminal MEP in the right limbs decreased and EEG showed no epileptic discharge. **K** A neuronavigation probe was used to detect the edge of the resection cavity and tracts in real time. **L** iMRI scanning. **M** iMRI showed a residual tumor located at the back of the cavity, close to the back of the putamen and the posterior limb of the internal capsule. The surgical plan was updated. **N** Removed residual tumor. **O** Pre, intra, and postoperative MRI. The final EOR was 100%. Pathology: anaplastic oligodendroglioma, WHO 3. At one week postoperatively, the muscle strength of the right upper and lower limbs was grade 4 and 3, respectively. At 3 and 6 months postoperatively, the muscle strength of the right limbs was grade 4 and 4.5, respectively. Radiotherapy plus concomitant and adjuvant TMZ chemotherapy were performed. No tumor recurrence or death occurred until the follow-up date, and PFS and OS were both 28.7 months

The resection corridor was created away from the language cortices according to neuronavigation, the protection of the language area was guided by neuronavigation under GA. If the brain drift was identified by the surgeon or the tumor was thought to be completely removed, iMRI was performed. The magnet was semiautomatically moved to the operating room through the rail. During the iMRI scanning, the anesthesiologist remotely observed the patient’s vital signs. If brain drift or residual tumor was detected by iMRI, the data were imported to iPlan and the surgical plan was updated. Further resection was performed according to neuronavigation and DES/IONM. After resection was completed, iMRI scanning was performed again to assess EOR and complications (hemorrhage, ischemia, etc.). During the whole procedure, multiple iMRI scanning can be performed to increase EOR and to correct brain drift whenever necessary so that the accuracy of neuronavigation can be maintained and language function can be precisely protected (Fig. 1).

### Postoperative treatment and follow-up

Patients with HGG were recommended to receive radiotherapy plus concomitant (60 Gy + TMZ 75 mg/m^2^/day) and adjuvant TMZ chemotherapy (150–200 mg/m^2^/day) [29, 30]. Regular MRI scanning was performed for patients every 3 months. The patients were followed up every 3 months, and the follow-up time was up to November 2021.

### Systematic review of previous studies

The detailed process was provided in Supplementary material 1. The rates of GTR, incidences of TLD and PLD were analyzed in a meta-analysis. The PFS and OS of previous studies were also reviewed and summarized. Our results were compared with the results of a meta-analysis of previous studies.

### Statistical analysis

Statistical analysis was performed by SPSS 21.0. Continuous parametric variables were compared between groups by Student’s *t* test. Categorical variables were compared between groups by the χ^2^ or Fisher’s exact test. Nonparametric variables were compared between groups by the Mann–Whitney *U* test. The Kaplan–Meier method was used to estimate and depict survival curves. Survival curves were compared between groups by the log-rank test. The significant difference was considered to exist between groups if a *P* value < 0.05.

Meta-analysis of previous studies was performed by STATA 14.0. The extent of heterogeneity among studies was evaluated by the Q test and the inconsistency index (*I*^2^). If *P* < 0.1 of Q test or *I*^2^ > 50%, heterogeneity was considered to be significant, then a random effects model was used to pool the incidence or GTR rate of previous studies. Otherwise a fixed effects model was used.

## Results

**Fig. 2:**
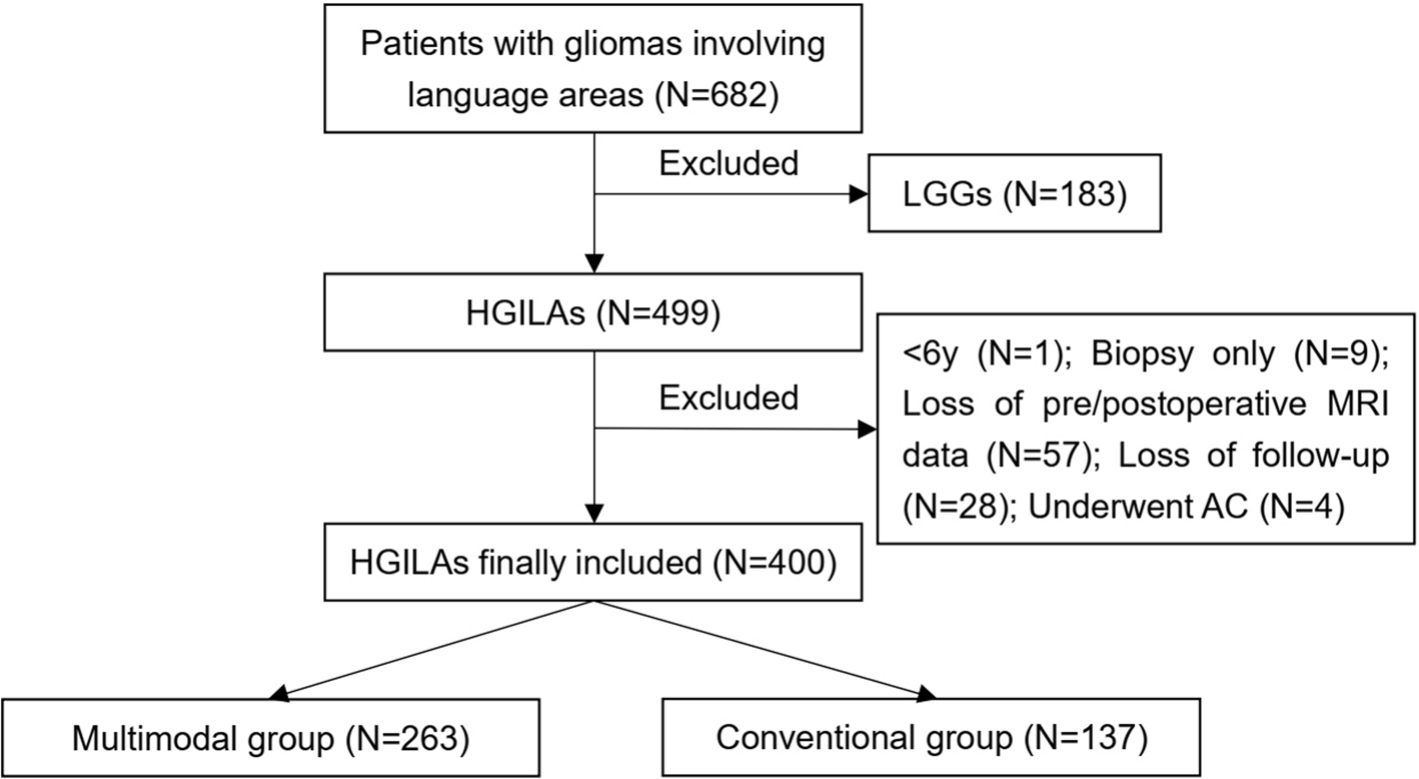
Process of patients’ selection

In total, there were 682 patients with glioma involving language areas. Finally, 400 patients with HGILAs who underwent surgery under GA were included, among which 263 patients belonged to multimodal group, 137 belonged to conventional group (Fig. 2).

### Comparison between multimodal and conventional group

**Fig. 3:**
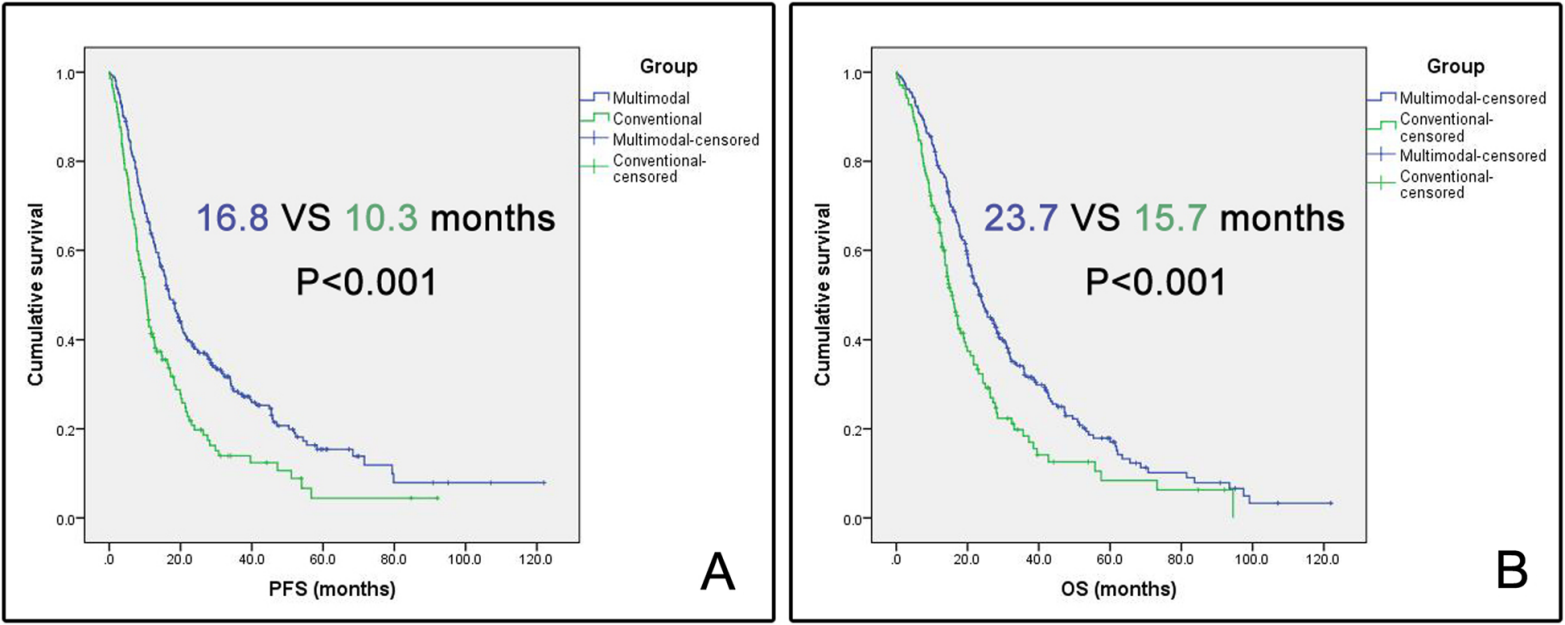
Survival curves of two groups. Blue and green curves represent the survival curves of multimodal group and conventional group respectively

The baseline clinical and tumor features were summarized in Table 1. The conventional group had the older age and shorter TMZ cycles than multimodal group. Other clinical and tumor features did not have significant differences between the two groups. The multimodal group had a higher median EOR than the conventional group (100% [IQR 98.57–100%] versus 94.32% [IQR 89.22–100%], *P* < 0.001) (Table 2). The rate of GTR of multimodal group was higher than that of conventional group (73.8% vs 36.5%, *P* < 0.001). The multimodal group had longer median operation time and hospital stay than the conventional group. The incidences of postoperative complications and seizures were not significantly different between two groups. The multimodal group had higher AQ and KPS than the conventional group at 1 day, 3 and 6 months postoperatively. The multimodal group had the lower incidences of TLD (17.1% versus 29.2%, *P* = 0.005) and PLD (9.5% versus 19.7%, *P* = 0.004) than the conventional group. The survival analysis demonstrated that the multimodal group had a longer PFS (16.8 versus 10.3 months, *P* < 0.001) and OS (23.7 versus 15.7 months, *P* < 0.001) than the conventional group. The survival curves were showed in Fig. 3.

**Table 1:** Baseline clinical and tumor features of HGILAs between the multimodal and conventional groups

Variables		Multimodal group (*N* = 263)	Conventional group (*N* = 137)	*P*
Male, *N* (%)		164 (62.4)	81 (59.1)	0.53
Age, Mean ± SD		47.9 ± 13.6	51.0 ± 13.9	**0.03**
AQ, Median (IQR)		87.0 (72.2–100)	87.9 (68.7–100)	0.86
Seizures, *N* (%)		59 (22.4)	30 (21.9)	0.98
KPS, median (IQR)		70 (60–80)	70 (60–80)	0.21
Recurrent glioma		57 (21.7)	26 (19.0)	0.53
Language cortices involved, *N* (%)		128 (48.7)	67 (48.9)	0.96
Tumor Volume, median (range)		46.02 (28.33–73.10)	52.31 (26.55–76.21)	0.85
Nearest distance to language areas, median (IQR)		1.20 (0–4.18)	1.21 (0–4.73)	0.64
WHO grade	3	100 (38.0)	42 (30.7)	0.14
	4	163 (62.0)	95 (69.3)	
*IDHmut*^a^		27 (33.3)	17 (37.8)	0.62
*MGMTmet*^a^		35 (43.2)	27 (60.0)	0.07
1p/19q loh^a^		11 (13.6)	4 (8.9)	0.44
*TERTmut*^a^		42 (51.9)	24 (53.3)	0.87
Radiotherapy, *N* (%)		206 (78.3)	100 (73.0)	0.23
TMZ cycles, median (IQR)		6 (3–12)	6 (0–6)	**0.01**

Boldface type indicated statistical significance

*IDHmut* *IDH* mutation, *MGMTmet* methylation of the *MGMT* promoter, *1p/19q loh* 1p/19q chromosome loss of heterozygosity, *TERTmut* *TERT* promoter mutation

^a^Molecular positive results/tests × 100%

**Table 2:** Comparison of outcomes between multimodal and conventional groups

Variables	Multimodal group (*N* = 263)	Conventional group (*N* = 137)	*P*
EOR (%), median (IQR)	100 (98.57–100)	94.32 (89.22–100)	** < 0.001**
GTR (EOR = 100%)	194 (73.8)	50 (36.5)	** < 0.001**
Operation time (hours), median (IQR)	7.75 (6.58–9.33)	5.50 (4.46–6.50)	** < 0.001**
Length of hospital stay (days), median (IQR)	17 (14–22)	16 (13–19.5)	**0.001**
Other complications, *N* (%)	16 (6.1)	13 (9.5)	0.21
Seizures, *N* (%)	30 (11.4)	12 (8.8)	0.41
AQ 1 day, median (IQR)	86.8 (75.0–100)	79.2 (61.4–100)	**0.008**
AQ within 3 months, median (IQR)	87.2 (75.4–100)	82.0 (61.2–100)	**0.01**
AQ within 6 months, median (IQR)	90.2 (76.2–100)	81.8 (57.6–100)	**0.006**
KPS 1 day, median (IQR)	70 (60–90)	70 (60–80)	**0.02**
KPS within 3 months, median (IQR)	80 (70–90)	80 (60–90)	**0.03**
KPS within 6 months, median (IQR)	80 (70–90)	90 (50–90)	**0.05**
Temporary language deficit (within 3 months)	45 (17.1)	40 (29.2)	**0.005**
Permanent language deficit (within 6 months)	25 (9.5)	27 (19.7)	**0.004**
Median PFS (95%CI)	16.8 (14.4–19.2)	10.3 (8.8–11.8)	** < 0.001**
Median OS (95%CI)	23.7 (21.0–26.4)	15.7 (13.4–18.0)	** < 0.001**

Boldface type indicated statistical significance

### Findings of multiple uses of iMRI

**Fig. 4:**
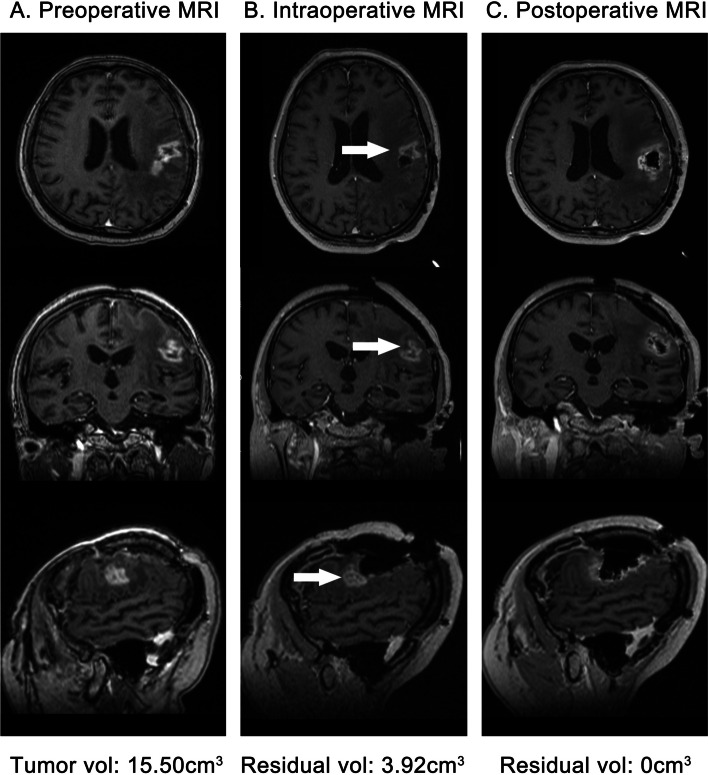
A case of using multiple iMRI to increase the EOR of HGILAs. The patient was a 54-year-old woman who had a recurrent tumor with pathologic diagnosis of GBM (WHO grade 4). The pre- (**A**), intra- (**B**), and postoperative (**C**) MRIs showed the residual tumor and further resection that increased the EOR from 74.71 to 100%. The arrows showed the residual tumor was located in the front of Broca area

In the multimodal group, 52 patients were identified as having residual tumors on the first iMRI, and further resections and multiple iMRI were performed. Their final median EOR was 100% (IQR 97.45–100%). The median EOR was 89.42% (83.30–94.25%) on the first iMRI scan. The EOR was significantly increased by 10.58% (*P* < 0.001) (Fig. 4).

### Systematic review of previous studies

There were 31 studies on the resection of GILAs [18, 31–60]. Among these studies there were 5 studies under GA and 14 studies under AC (Supplementary Table 1). There were also 12 studies that compared AC with GA. There were only 6 studies that included patients with HGG alone [33, 34, 36, 37, 48, 58], other studies also included LGG. Nine studies used intraoperative multimodal techniques under AC [38, 43–45, 47, 53, 54, 57, 59] and 5 studies used multimodal techniques under GA [33, 34, 53, 54, 59]. The results of previous studies of HGG were compared to our results. Our multimodal group achieved a higher median EOR and a relative high GTR rate compared to cohorts in previous studies. Our cohort had a relative high incidence of PLD. The median PFS and OS of our cohort were both longer than cohorts in previous studies (Table 3).

**Table 3:** Compare our cohort with previous studies of HGG

Studies	Design	*N*	GA or AC	Multimodal techniques	EOR (%)	GTR	Temporary language deficit	Permanent language deficit	Median PFS (95%CI)	Median OS (95%CI)
Our study	Retro	263	GA	YES	100 (98.57–100)^a^	73.8%	17.1%	9.5%	16.8 (14.4–19.2)	23.7 (21.0–26.4)
Chen 2017^c^ [33]	Retro	51	GA	YES	96 (86–100)^a^	31.4%	15.7%	3.9%	18 (9–42)	28 (14–49)
Schucht 2012 [58]	Retro	69	GA	NO	NR	76.8%	NR	10.1%	NR	NR
Nakajima 2019 [48]	Case control	30	GA	NO	96% ± 9.1%^b^	NR	23.3%	NR	NR	NR
		30	AC	NO	97% ± 8.7%^b^	NR		NR	NR	NR
Gerritsen 2019 [37]	Case control	111	GA	NO	79.7%^a^	NR	26.8%	5.4%	NR	15 (1318)
		37	AC	NO	100%^a^	NR	10.8%	0	NR	17 (12–36)
Feigl 2010 [36]	Pros	18	GA	NO	NR	64%	0	NR	NR	NR
D’Andrea 2015^c^ [34]	Retro	27	GA	YES	NR	77.8%	59.3%	11.1%	NR	17 (10.2–23.8)

^a^Median

^b^Mean

^c^Used multimodal techniques

**Fig. 5:**
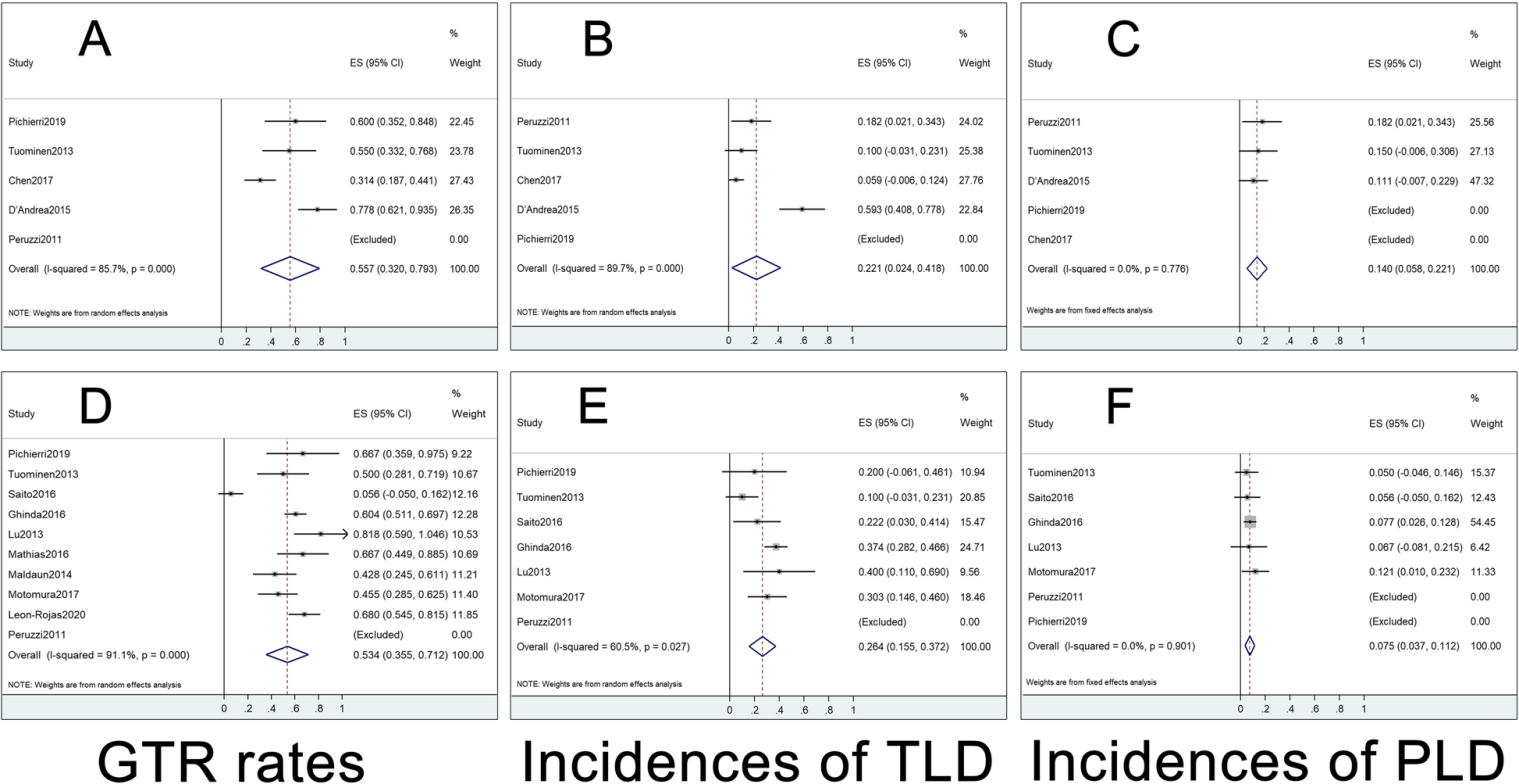
Forest plots of previous multimodal studies under GA (**A**–**C**) and AC (**D**–**F**) respectively. Left column: rates of GTR. Middle column: incidences of TLD. Right column: incidences of PLD

We also compared our multimodal cohort with cohorts of previous studies that used multimodal techniques under GA or AC (Table 4). The median EOR of our multimodal group was higher than cohorts in previous studies. Meta-analysis was performed on the GTR, TLD, and PLD of cohorts in previous studies (Fig. 5). The results showed that our multimodal group achieved a higher GTR rate (73.3%) than cohorts in previous studies under GA (55.7% [95%CI 32.0–79.3%]) or AC (53.4% [95%CI 35.5–71.2%]). Our multimodal group also achieved a lower incidence of TLD than cohorts in previous studies using multimodal techniques (18.1% vs 22.1% [95%CI 2.4–41.8%] of GA vs 26.4% [95%CI 15.5–37.2%] of AC). Our multimodal group achieved similar incidence of PLD with cohorts in previous GA studies (13.8% vs 14.0% [95%CI 5.8–22.1%]); however, it was higher than cohorts in AC studies (13.8% vs 7.5% [95%CI 3.7–11.2%]). Three studies reported the survival and only 2 studies included HGG alone [33, 34]. Our multimodal group achieved a relative high median PFS and OS.

**Table 4:** Compare our cohort with previous studies used multimodal techniques under GA or AC

Studies	Design	*N*	GA or AC	Only HGG	EOR (%)	GTR	Temporary language deficit	Permanent language deficit	Median PFS (95%CI)	Median OS (95%CI)
Our study	Retro	263	GA	Yes	100 (98.57–100)^a^	73.8%	17.1%	9.5%	16.8 (14.4–19.2)	23.7 (21.0–26.4)
Peruzzi 2011 [53]	Case control	22	GA	No, HGG = 19	NR	100%	18.2%^c^	18.2%^c^	NR	NR
		22	AC	No, HGG = 20	NR	100%	0^c^	0^c^	NR	NR
Pichierri 2019 [54]	Case control	26	GA	No, HGG = 15	NR	60%	0^c^	0^c^	NR	NR
		20	AC	No, HGG = 9	NR	66.7%	20%^c^	0^c^	31^c^	NR
Tuominen 2013 [59]	Case control	20	GA	No, HGG = 12	NR	55.5%^c^	10%^c^	15%^c^	NR	NR
		20	AC	No, HGG = 13	NR	50%^c^	10%^c^	5%^c^	NR	NR
Chen 2017 [33]	Retro	51	GA	Yes	96 (86–100)^a^	31.4%	15.7%	3.9%	18 (9–42)	28 (14–49)
D’Andrea 2015 [34]	Retro	27	GA	Yes	NR	77.8%	59.3%	11.1%	NR	17 (10.2–23.8)
Saito 2016 [57]	Retro	18	AC	No, HGG = 9	90% ± 7.1%^bc^	5.6%^c^	22.2%^c^	5.6%^c^	NR	NR
Ghinda 2016 [38]	Retro	106	AC	No, HGG = 42	96.4% ± 9.1%^b^	60.4%^c^	37.4%^c^	7.7%^c^	NR	NR
Lu 2013 [44]	Pros	30	AC	No, HGG = 11	100 (73.9–100)^ac^	81.8%	40%^c^	6.7%^c^	NR	NR
Mathias 2016 [46]	Retro	18	AC	No, HGG = 8	NR	66.7%^c^	NR	NR	NR	NR
Maldaun 2014 [45]	Retro	42	AC	No, HGG = 28	90^ac^	42.8%	NR	NR	NR	NR
Motomura 2017 [47]	Retro	33	AC	No, HGG = 11	NR	45.5%^c^	30.3%^c^	12.1%^c^	NR	NR
Leon-Rojas 2020 [43]	Pros	46	AC	No, HGG = 28	NR	68%^c^	NR	NR	NR	NR
Meta-analysis of GA	/	/	/	/	/	55.7% (95%CI 32.0–79.3%)^c^	22.1% (95%CI 2.4–41.8%)^c^	14.0% (95%CI 5.8–22.1%)^c^	/	/
Meta-analysis of AC	/	/	/	/	/	53.4% (95%CI 35.5–71.2%)^c^	26.4% (95%CI 15.5–37.2%)^c^	7.5% (95%CI 3.7–11.2%)^c^	/	/

^a^Median, ^b^Mean, ^c^Calculated by total patients of LGG and HGG

## Discussion

Neurological function protection is an important factor that should be considered in the resection of gliomas involving eloquent areas. Gogos 2020 summarized the continuous innovation and improvement of AC combined with DEC/IONM for brain mapping and believed it was the gold standard method for glioma surgery involving eloquent areas [8]. The Berger and Duffau teams have conducted many clinical practice examples and studies of glioma under AC and established the standard method of brain mapping under AC [61, 62]. However, an increasing number of studies have indicated that not all gliomas involving eloquent areas need to be removed under AC. Our center began to use neuronavigation in surgery in 2002 and has used intraoperative multimodal techniques since 2009. After years of clinical practice, we formed a mature method of multimodal techniques-assisted surgery for HGILAs under GA. Therefore, it was thought that some HGILAs can also achieve maximal safe resection under GA based on our experience.

The EOR and GTR rates of multimodal group were higher than those of conventional group (only neuronavigation was used), which fully demonstrated the effect of DES/IONM and iMRI on increasing EOR under GA. In addition, multiple uses of iMRI and further resection increased the EOR by 10.58% in our study. Previous studies have demonstrated an approximately 10% increase in EOR because of multiple uses of iMRI, which was slightly lower than our result [45, 63]. Multimodal group had the higher AQs than conventional group at different time points postoperatively. In addition, multimodal group had lower incidences of TLD and PLD than conventional group. These results demonstrated the protective effect of multimodal techniques for language function. The higher EOR and better functional outcomes also caused longer survival in the multimodal group. Language function still improved at 6 months postoperatively in all patients in both groups, which indirectly proved language plasticity. Many other previous studies have reported evidence of language function plasticity after glioma resection [64, 65]. All these results provide a foundation for maximal safe resection of GILAs under GA. The postoperative time points of permanent neurological deficits ranged from 2 weeks to 6 months among previous studies. According to De Witt Hamer 2012, we defined the time point of TLD as within 3 months [66]. We thought 6 months was a more appropriate time point for PLD because of the continuous improvement of language and KPS until 6 months postoperatively in our study.

Because the language network contained many cortical and subcortical structures, the HGILAs cannot be defined clearly in previous studies. If all these structures were included to define HGILAs, most patients with gliomas would met the definition and be included in the cohort of HGILAs. To reduce the scope of patients’ inclusion, we referred to the classical definition of language areas and proposed our definition of HGILAs in this manuscript. Our language areas mainly included traditional language regions (Broca and Wernicke areas, dorsal premotor cortex and arcuate fasciculus). Other specific language regions and fibers (right fusiform gyrus, anterior cingulate cortex, superior longitudinal fasciculus, etc.) were not considered when we defined the HGILAs. Direct electrical stimulation identified that the safe distance was more than 1 cm between resection edge and language regions in most classical studies [22–24]. In this study, we defined GILAs as the glioma within 2 cm of language areas on preoperative MRI to include more patients which was reasonable.

The definitions of GTR varied among previous studies, and most studies defined EOR = 100% or ≥ 98% as GTR. We defined EOR of 100% as GTR in our cohort. The rate of GTR of our multimodal group was higher than the meta-analysis results of previous studies used multimodal techniques under GA or AC. Our multimodal group also achieved a lower incidence of TLD, but the incidence of PLD of our multimodal group was a little higher than the meta-analysis result of previous studies of AC. We thought the reason was that some studies did not only include GILAs but also may include gliomas of other eloquent areas. Such as Peruzzi 2011 [53], Pichierri 2019 [54], Tuominen 2013 [59], Ghinda 2016 [38], Mathias 2016 [46], Maldaun 2014 [45], Motomura 2017 [47] and Leon-Rojas 2020 [43], although these studies reported the incidences of TLD and PLD, the results may not reflect the real incidences because the inclusion of gliomas of other eloquent areas. The median PFS and OS of our cohort was longer than those of most cohorts in previous studies. Although the survival of Chen 2017 was longer than our cohort, we thought it was because it also included insular HGG of non-dominant hemisphere. Therefore, we thought surgery assisted by multimodal techniques under GA can also achieve maximal safe resection for HGILAs.

The choice of GA or AC for HGILAs is a clinical problem that needs to be explored. Rossi 2022 used clinical and imaging variables to design a motor mapping score, which was then applied in the choice of AC or GA for the resection of perirolandic glioma in the nondominant hemisphere. However, it only designed for glioma involving motor areas, and the variables were selected based on experience, not all clinical and tumor features were considered for analysis, furthermore multivariate analysis was not performed to identify significant variables [67]. In this study, we found that surgery assisted by multimodal techniques can achieve maximal safe resection under GA while its incidence of permanent language deficit was a little higher than that of meta-analysis of previous AC studies (9.5% versus 7.5% [3.7–11.2%]). We speculate that a choice model can be established based on the clinical and tumor features to predict the probability of TLD/PLD. Then, according to this probability we can classify patients of HGILAs into GA and AC group. Thus not all patients need AC, some patients of HGILAs can be performed surgery assisted by multimodal techniques under GA. This work is being done based on our HGILAs cohort in another paper.

Some limitations existed in this study. (1) Retrospective studies have inherent limitations that may cause some bias. (2) Different study designs, different intraoperative techniques used, GTR definitions and time points of PLD caused some heterogeneity and bias of meta-analysis results. While the meta-analysis results can present the efficacy of AC in a certain extent.

## Conclusions

Surgery assisted by multimodal techniques can also achieve maximal safe resection for patients with HGILAs under GA. The EOR can be increased significantly in parallel with the protection of language function. Further prospective studies are needed to compare GA with AC for HGILAs, a choice model are needed to be established to help different patients choose the most suitable strategy of surgery.

### Supplementary Information

**Additional file 1: Supplementary material 1.** PubMed and Embase were searched for studies of GA or AC in GILAs (from January 2000 to January 2021) using the following keywords: “glioma”; “eloquent” or “function” or “functional” or “language”; and “awake” or “general anesthesia”.

**Additional file 2: Supplementary Table 1.** Summary of previous studies about GA or AC in glioma resection.

## Data Availability

The data that support the findings of our study are available from the corresponding author upon reasonable request.

## Abbreviations

AC: Awake craniotomy
DES: Direct electrical stimulation
GA: General anesthesia
GILAs: Gliomas involving language areas
HGILAs: High-grade gliomas involving language areas
iMRI: Intraoperative magnetic resonance imaging
IONM: Intraoperative neuromonitoring
TLD/PLD: Temporary/permanent language deficit
