# Supplementary material for: Resection of high-grade glioma involving language areas assisted by multimodal techniques under general anesthesia: a retrospective study
Source: Chin Neurosurg J. 2023 Sep 11;9:25. doi: 10.1186/s41016-023-00340-5 (PMC10494413; doi:10.1186/s41016-023-00340-5)
Supplement: Supplementary file 1 — Additional file 1: Supplementary material 1. PubMed and Embase were searched for studies of GA or AC in GILAs (from January 2000 to January 2021) using the following keywords: “glioma”; “eloquent” or “function” or “functional” or “language”; and “awake” or “general anesthesia”. [file 41016_2023_340_MOESM1_ESM.docx]

**Supplementary material 1**

PubMed and Embase were searched for studies of GA or AC in GILAs (from January 2000 to January 2021) using the following keywords: “glioma”; “eloquent” or “function” or “functional” or “language”; and “awake” or “general anesthesia”. We performed quality assessment for the included studies according to common meta-analysis methods and PRISMA criteria. STATA 14.0 was used to perform the meta-analysis of rates of different variables in previous studies. We used the Q test and the inconsistency index (I^2^) to evaluate the extent of heterogeneity in each study. Heterogeneity was considered to be significant if P<0.1 or I^2^>50%. In this case, the rates were pooled using a random-effects model. Otherwise, a fixed-effects model was used.

PUBMED 2000-01-01——2021.12.31, limited in English

("Glioma"[Mesh] OR glioma[tw] OR glioblastom*[tw] OR astrocytom*[tw] OR oligodendrogliom*[tw] OR oligoastrocytom*[tw])

AND ("language"[Mesh] OR language[tw] OR language function[tw] OR language area[tw] OR eloquent[tw] OR Broca[tw] OR Wernicke[tw] OR arcuate tract[tw])

AND (awake[tw] OR awake craniotomy[tw] OR awake surgery[tw] OR general anesthesia[tw] OR mapping[tw] OR map[tw])

EMBASE

“glioma”/exp OR “glioma”:ab,ti OR glioblastom*:ab,ti OR astrocytom*:ab,ti OR oligodendrogliom*:ab,ti OR oligoastrocytom*:ab,ti

AND ("language"/exp OR "language function"/exp OR "language area"/exp OR "eloquent":ab,ti OR "Broca":ab,ti OR "Wernicke":ab,ti OR " arcuate tract":ab,ti)

AND (“awake”:ab,ti OR “awake craniotomy”:ab,ti OR “awake surgery”:ab,ti OR “general anesthesia”:ab,ti OR “mapping”:ab,ti OR “map”:ab,ti)

Limited: 2000-2021, english

PUBMED724 EMBASE856
