# Supplementary material for: Resection of high-grade glioma involving language areas assisted by multimodal techniques under general anesthesia: a retrospective study
Source: Chin Neurosurg J. 2023 Sep 11;9:25. doi: 10.1186/s41016-023-00340-5 (PMC10494413; doi:10.1186/s41016-023-00340-5)
Supplement: Supplementary file 2 — Additional file 2: Supplementary Table 1. Summary of previous studies about GA or AC in glioma resection. [file 41016_2023_340_MOESM2_ESM.docx]

Supplementary table 1. Summary of previous studies about GA or AC in glioma resection

| **Study** | **Design** | **N** | **Sex (Male [%]）** | **Mean or median age** | **Areas involved by tumor** | **Pathology** | | **Anesthesia** | | **DES** | **IONM** | **Neuronavigation** | **iMRI** | **EOR** | **Incidence of TMD/PMD** | **Incidence of TLD/PLD** | | **Pre/intra/ post-operative seizure** | **Pre/post- operative KPS** | **complications** | **Median PFS** | **Median OS** |
| --- | --- | --- | --- | --- | --- | --- | --- | --- | --- | --- | --- | --- | --- | --- | --- | --- | --- | --- | --- | --- | --- | --- |
|  |  |  |  |  |  | **LGG** | **HGG** | **GA** | **AC** |  |  |  |  |  |  |  |  |  |  |  |  |  |
| **Peraud2004** | Retrospective | 14 | 6 (42.9) | 31.1±10.1 (8-50) | Insular, Supramarg. | 11 | 3 | 0 | 14 | YES | NO | NO | NO | GTR=50% | 50.0%/14.3% | 57.1%/14.3% | | 92.9%/NA/42.9% | NA | NA | NA | NA |
| **Peruzzi2011** | Case control | 22 | 16 (72.7) | 50.5# (27-65) | Unspecified | 3 | 19 | 22 | 0 | YES | YES | YES, 22 | YES, 8 | GTR=100% | 9.1%/0 | 18.2%/18.2% | | NA | NA | NA | NA | NA |
|  |  | 22 | 15 (68.2) | 50.7# (36-69) |  | 2 | 20 | 0 | 22 | YES | YES | YES, 22 | YES, 2 | GTR=100% | 13.6%/4.5% | 0/0 | |  |  |  |  |  |
| **Sacko2011†** | Prospective | 72 | 34 (47.3) | 46.3# | Eloquent areas | 14 | 31 | 72 | 0 | NO | YES | YES | NO | GTR=14% | 30.5%/NA | 27.7%/NA | | NA/NA/8.3% | NA | 6.9% | NA | GBM=13 LGG=56 |
|  |  |  |  |  |  |  |  |  |  |  |  |  |  |  | Neurological deficits =81.9%/15.3% | | |  |  |  |  |  |
|  |  | 214 | 114 (53.3) | 46.5# |  | 72 | 71 | 0 | 214 | YES | YES | YES | NO | GTR=37% | 6.0%/NA | 11.6%/NA | | NA/NA/4.6% | NA | 3.7% | NA | GBM=22 |
|  |  |  |  |  |  |  |  |  |  |  |  |  |  |  | Neurological deficits =24.3%/4.6% | | |  |  |  |  |  |
| **Pichierri2019** | Case control | 26 | 15 (57.7) | 44±16 | Eloquent areas=22 | 11 | 15 | 26 | 0 | YES | YES | YES | YES | GTR=53.8%  HGG=60% | 3.8%/0 | 0/0 | | NA/0/0 | NA | 3.8% | 5-year 68% | 5-year 76.9% |
|  |  | 20 | 14 (70.0) | 46±14 | Eloquent areas | 11 | 9 | 0 | 20 | YES | YES | YES | YES | GTR=60%  HGG=66.7% | 10%/0 | 20%/0 | | NA/5%/0 | NA | 0 | 31 | 5-year 90% |
| **Zelitzki2019†** | Retrospective | 41 | 24 (58.5) | 50.5±17.1 | Motor areas | 10 | 24 | 41 | 0 | YES | YES | YES | NO | 91.1±11.8%, GTR=41.4% | 29.3%/12.2% | 9.8%/7.3% | | 31.7%/NA/7.3% | 93.4±9.9/NA | 7.3% | NA | NA |
|  |  | 44 | 27 (61.4) | 47±15.9 | Motor areas | 15 | 26 | 0 | 44 | YES | YES | YES | NO | 86.2±18.4%, GTR=40.9% | 13.6%/9.1% | 20.5%/9.1% | | 72.3%/NA/2.3% | 94.5±7.3/NA | 2.3% | NA | NA |
| **Eseonu2017** | Case control | 31 | 19 (61.3) | 50.3±17.1 | Perirolandic areas | 7 | 24 | 31 | 0 | YES | YES | YES | NO | GTR=6.5% | 38.7%/9.7% | 6.5%/6.5% | | 64.5%/16.1%/29% | 81.9±13.5/81.1±21.1 | 9.7% | NA | NA |
|  |  | 27 | 19 (70.4) | 49.1±14.9 |  | 4 | 23 | 0 | 27 | YES | YES | YES | NO | GTR=25.9% | 59.3%/11.1% | 0/14.8% | | 85.2%/7.4%/33.3% | 84.8±11.6/93.3±11.9 | 3.7% | NA | NA |
| **Study** | **Design** | **N** | **Sex (Male [%]）** | **Mean or median age** | **Areas involved by tumor** | **Pathology** | | **Anesthesia** | | **DES** | **IONM** | **Neuronavigation** | **iMRI** | **EOR** | **Incidence of TMD/PMD** | | **Incidence of TLD/PLD** | **Pre/intra/ post-operative seizure** | **Pre/post- operative KPS** | **complications** | **Median PFS** | **Median OS** |
|  |  |  |  |  |  | **LGG** | **HGG** | **GA** | **AC** |  |  |  |  |  |  |  |  |  |  |  |  |  |
| **Gravesteijn2017** | Retrospective | 28 | 18 (64) | 49.0±15.1 | Insular | 13 | 15 | 28 | 0 | YES | NA | YES | NO | NA | Neurological deficits=57%/48% | | | 71.4%/NA/NA | NA | 0 | NA | 5-year 28.6%，GBM5-y 6.7% |
|  |  | 24 | 20 (83.3) | 41.2±10.8 |  | 16 | 8 | 0 | 24 | YES | NA | YES | NO | NA | Neurological deficits=67%/33% | | | 58.3%/NA/NA | NA | 8.3% | NA | 5-year 66.7%，GBM5-y 0% |
| **Gupta2007†** | RCT | 27 | 20 (74.1) | 41.3±17.3 | Language or sensory/ motor cortices | 15 | 7 | 27 | 0 | YES | YES | NO | NO | GTR=37% | 10.5%/11.8% | 25%/33.3% | | 81.5%/0/NA | 90 (70-90)/90 (70-100)* | NA | NA | NA |
|  |  | 26 | 20 (76.9) | 42.7±15.8 |  | 16 | 8 | 0 | 26 | YES | YES | NO | NO | GTR=38.5% | 38.9%/18.8% | 50%/42.9% | | 80.8%/3.8%/NA | 90 (50-90)/80 (50-100)* | NA | NA | NA |
| **Pinsker2007†** | Retrospective | 27 | NA | 50.3# | Language or sensory/ motor areas | 0 | 12 | 27 | 0 | NO | NO | YES | NO | GTR=33% | NA | NA | | NA | NA | NA | NA | NA |
|  |  | 52 | NA | 66.5# |  | 11 | 41 | 0 | 52 | YES | YES | YES | NO | GTR=72% | 7.7%/7.7% | 7.7%/0 | | NA/0/NA | NA | NA | NA | NA |
| **Tuominen2013** | Case control | 20 | 11 (55) | 43 (11-69)# | Language or motor areas | 8 | 12 | 20 | 0 | NO | NO | YES | YES | GTR=55% | 10%/20% | 10%/15% | | 75%/0/NA | NA | NA | NA | NA |
|  |  | 20 | 11 (55) | 44 (16-67)# |  | 7 | 13 | 0 | 20 | YES | YES | YES | YES | GTR=50% | 5%/5% | 10%/5% | | 85%/10%/NA | NA | NA | NA | NA |
| **Lang2001** | Retrospective | 17 | 3 (17.6) | 42.5# | Insular | 6 | 11 | 17 | 0 | NA | YES | NO | NO | 91% (70-99%)* | 17.6%/17.6% | 11.8%/0 | | NA | NA | NA | NA | NA |
|  |  | 5 | 3 (60) | 37.6# |  | 3 | 2 | 0 | 5 | YES | YES | NO | NO | 75% (38.5-87%)* | 20%/0 | 80%/0 | | NA | NA | NA | NA | NA |
| **Study** | **Design** | **N** | **Sex (Male [%]）** | **Mean or median age** | **Areas involved by tumor** | **Pathology** | | **Anesthesia** | | **DES** | **IONM** | **Neuronavigation** | **iMRI** | **EOR** | **Incidence of TMD/PMD** | **Incidence of TLD/PLD** | | **Pre/intra/ post-operative seizure** | **Pre/post- operative KPS** | **complications** | **Median PFS** | **Median OS** |
|  |  |  |  |  |  | **LGG** | **HGG** | **GA** | **AC** |  |  |  |  |  |  |  |  |  |  |  |  |  |
| **Chen2017** | Retrospective | 51 | 32 (62.7) | 54.2 (23-69)# | Insular | 0 | 51 | 51 | 0 | YES | YES | YES | YES | 96% (86-100%)*, GTR =31.4% | 5.9%/3.9% | 5.9%/0 | | 70.6%/NA/NA | 90 (50-100)*/90 (70-100)* | 0 | 18 (9-42)* | 28 (14-49)* |
|  |  |  |  |  |  |  |  |  |  |  |  |  |  |  | Neurological deficits =15.7%/3.9% | | |  |  |  |  |  |
| **Schucht2012** | Retrospective | 69 | 41 (59.4) | 59.5 (38-80)# | Unspecified | 0 | 69 | 69 | 0 | YES | YES | YES | NO | GTR=76.8% | NA/7.2% | NA/0 | | NA | 82.9/83.9# | NA | NA | NA |
|  |  |  |  |  |  |  |  |  |  |  |  |  |  |  | Neurological deficits=NA/10.1% | | |  |  |  |  |  |
| **Pastor2013** | Prospective | 34 | 19 (55.9) | 49.8±2.4 | Eloquent areas | 6 | 30 | 34 | 0 | YES | YES | YES | NO | 95.1%#, GTR(>98%)=66.7% | 8.3%/0 | 2.8%/0 | | 25%/NA/0 | NA | 5.6% | NA | NA |
|  |  |  |  |  |  |  |  |  |  |  |  |  |  |  | Neurological deficits=16.7%/0 | | |  |  |  |  |  |
| **Saito2016** | Retrospective | 18 | 9 (50) | 39 (19-63)# | Broca area | 9 | 9 | 0 | 18 | YES | YES | YES | YES | 90%±7.1%#, GTR=5.6% | NA | 22.2%/5.6% | | NA | NA | NA | NA | NA |
| **Brennum2018** | Retrospective | 92 | 43 (46.7) | 36 (15-68)# | Unspecified | 73 | 19 | 0 | 92 | YES | YES | YES | NO | 92% (21-100%)* | Neurological deficits=33%/9% | | | 78%/NA/22% | 90 (80-100)/90 (82.5-100)* | NA | NA | NA |
| **Ghinda2016** | Retrospective | 106 | 74 (69.8) | 41.7±12.5 (18-76) | Eloquent areas | 64 | 42 | 0 | 106 | YES | YES | YES | YES | 100%*, GTR=60.4%LGG=89.1%±19.6%#, HGG=96.4%±9.1%# | 20.7%/4.3% | 37.4%/7.7% | | 52.8%/3.8%/NA | NA | NA | NA | NA |
|  |  |  |  |  |  |  |  |  |  |  |  |  |  |  | Neurological deficits =46.2%/8.7% | | |  |  |  |  |  |
| **Lu2013** | Prospective | 30 | 21 (70) | 45.5 (19-67)* | Language areas | 19 | 11 | 0 | 30 | YES | YES | YES | YES | 100% (73.9-100%)*, GTR=60%, LGG GTR=47.4%, HGG GTR =81.8% | NA | | 40%/6.7% | 53.3%/13.3%/NA | NA | 0 | NA | NA |
| **Study** | **Design** | **N** | **Sex (Male [%]）** | **Mean or median age** | **Areas involved by tumor** | **Pathology** | | **Anesthesia** | | **DES** | **IONM** | **Neuronavigation** | **iMRI** | **EOR** | **Incidence of TMD/PMD** | **Incidence of TLD/PLD** | | **Pre/intra/ post-operative seizure** | **Pre/post- operative KPS** | **complications** | **Median PFS** | **Median OS** |
|  |  |  |  |  |  | **LGG** | **HGG** | **GA** | **AC** |  |  |  |  |  |  |  |  |  |  |  |  |  |
| **Mathias2016** | Retrospective | 18 | 12 (66.7) | 42.5 (29-57)# | Eloquent areas | 8 | 8 | 0 | 18 | YES | YES | NO | YES | GTR(≥95%)=66.7% | Neurological deficits=20%/0 | | | 66.7%/NA/NA | NA | NA | NA | NA |
| **Maldaun2014** | Retrospective | 42 | 25 (59.5) | 41.2 (22-70)* | Motor, sensory, language cortices | 14 | 28 | 0 | 42 | YES | YES | YES | YES | 90%*, GTR (≥95%)=40.5%, LGG GTR(≥95%)=35.7%, HGG GTR(≥95%)=42.8% | Neurological deficits =26.2%/2.4% | | | NA/7.1%/NA | NA | NA | NA | NA |
| **Nakajima2018** | Retrospective | 66 | 39 (59.1) | 43 (16-73)* | Unspecified | 24 | 42 | 0 | 66 | YES | YES | NO | NO | GTR=40.9%, LGG GTR=29.2%, HGG GTR=47.6% | 21.2%/6.1% | 4.6%/1.5% | | NA | ≥70=100%/≥70=91.3% | NA | NA | NA |
| **Nakajima2019** | Case control | 30 | 19 (63.3) | 58.8±9.4 | Eloquent areas | 0 | 30 | 30 | 0 | YES | YES | NO | NO | 96%±9.1% | 43.3%/NA | 23.3%/NA | | NA | 90 (60-100)/70 (30-100)* | NA | NA | NA |
|  |  | 30 | 20 (66.7) | 53.2±11.7 |  | 0 | 30 | 0 | 30 | YES | YES | NO | NO | 97%±8.7% |  |  |  | NA | 90 (60-100)/80 (30-10)* | NA | NA | NA |
| **Pallud2017** | Prospective | 107 | 59 (55.1) | 40.8±12.5 (16-74) | Eloquent areas | 49 | 58 | 0 | 107 | YES | YES | NO | NO | LGG=86.3%±22.6%, WHO III=78.7%±30.4%, GBM=89%±29.5% | Neurological deficits =29.9%/1.9% | | | 79.4%/1.9%/90.2% | 95 (50-100)/91.8 (40-100)# | NA | PR=21, STG=44.5, GTR=53 | LGG=72, GBM=26 |
| **Study** | **Design** | **N** | **Sex (Male [%]）** | **Mean or median age** | **Areas involved by tumor** | **Pathology** | | **Anesthesia** | | **DES** | **IONM** | **Neuronavigation** | **iMRI** | **EOR** | **Incidence of TMD/PMD** | **Incidence of TLD/PLD** | | **Pre/intra/ post-operative seizure** | **Pre/post- operative KPS** | **complications** | **Median PFS** | **Median OS** |
|  |  |  |  |  |  | **LGG** | **HGG** | **GA** | **AC** |  |  |  |  |  |  |  |  |  |  |  |  |  |
| **Gerritsen2019** | Case control | 111 | 72 (64.9) | 48.3±14 | Eloquent areas | 0 | 111 | 111 | 0 | NO | NO | YES | NO | 79.7%* | 31.5%/15.3% | 26.1%/5.4% | | NA | 89.3±9.9/NA | NA | NA | 15 (95%CI: 13-18) |
|  |  |  |  |  |  |  |  |  |  |  |  |  |  |  | Neurological deficits =68.4%/23.4% | | |  |  |  |  |  |
|  |  | 37 | NA | 45.7±15.1 |  | 0 | 37 | 0 | 37 | YES | YES | YES | NO | 100%* | 10.8%/8.1% | 10.8%/0 | | NA | 89.7±11.2/NA | NA | NA | 17 (95%CI: 12-36) |
|  |  |  |  |  |  |  |  |  |  |  |  |  |  |  | Neurological deficits =43.2%/8.1% | | |  |  |  |  |  |
| **Lau2017** | Retrospective | 451 | 272 (60.3) | 42.3# | Motor or language areas | 215 | 236 | 0 | 451 | YES | YES | YES | NO | NA | 3.3%/2.4% | 5.1%/2.5% | | NA | NA | NA | NA | NA |
|  |  |  |  |  |  |  |  |  |  |  |  |  |  |  | Neurological deficits=8.4%/4.9% | | |  |  |  |  |  |
| **Motomura2017** | Retrospective | 33 | 25 (75.8) | 41 (28-67)* | Eloquent areas | 19 | 11 | 0 | 33 | YES | YES | YES | YES=25 | ≥90%=45.5% | 12.1%/0 | 30.3%/12.1% | | NA | NA | 3% | NA | NA |
| **Hervey-Jumper2015†** | Retrospective | 611 | NA | 43 (13-84)* | Motor, sensory, language areas | 259 | 335 | 0 | 611 | YES | YES | NO | NO | NA | Neurological deficits =9.5%/2.6% | | | 69.9%/3.3%/NA | ≥80=60.2%/NA | 2.1% | NA | NA |
| **Chacko2013†** | Retrospective | 67 | 55 (82.1) | 34.6 (13-58)* | Eloquent areas | 34 | 30 | 0 | 67 | YES | YES | NO | NO | GTR=65.7% | 10.4%/6% | 3%/0 | | NA/4.4%/NA | NA | 6% | NA | NA |
|  |  |  |  |  |  |  |  |  |  |  |  |  |  |  | Neurological deficits=13.4%/6% | | |  |  |  |  |  |
| **Leon-Rojas2020†** | Prospective | 46 | 26 (56.5) | 52 (26-78)* | Eloquent areas=76.5% | 9 | 28 | 0 | 46 | YES | YES | YES | YES | ≥95%=68% | Neurological deficits=28%/12% | | | NA/0/NA | NA | NA | NA | NA |
| **Feigl2010** | Prospective | 18 | 12 (66.7) | 55 (22-76)# | Eloquent areas | 0 | 18 | 18 | 0 | YES | YES | YES | NO | GTR=64% | 11.1%/NA | 0/NA | | 22.2%/0/5.6% | 90/89# | 0 | NA | NA |
| **D’Andrea2015** | Retrospective | 27 | 15 (55.6) | 65.8 | Language areas | 0 | 27 | 27 | 0 | NO | NO | YES | YES | GTR=77.8% | NA | 59.3%/11.1% | | NA | NA | NA | NA | 17.0 (95%CI: 10.2-23.8) |

*Median, #Mean, †Other brain tumors were included besides glioma. TMD/PMD, Temporary/permanent motor deficit. TLD/PLD, Temporary/permanent language deficit.
